# Perioperative Systemic Analgesia in Patients With Inflammatory Skin Disease: A Matched Cohort Study

**DOI:** 10.7759/cureus.115900

**Published:** 2026-09-07

**Authors:** Tiffany A Kumala, Tristan Nguyen, Misha V Koshelev

**Affiliations:** 1 Dermatology, University of Texas Health Science Center at Houston, Houston, USA; 2 Dermatology, University of Texas Medical Branch at Galveston, Galveston, USA

**Keywords:** analgesics, dermatology, electronic health records, inflammatory dermatoses, inflammatory skin disease, opioids, pain management, perioperative care, postoperative pain, propensity score matching

## Abstract

Background: Chronic inflammatory dermatoses are associated with altered nociception and increased opioid use in nonsurgical settings, but perioperative analgesic prescribing in this population has not been well characterized. We compared perioperative analgesic use, recorded postoperative pain, and surgical outcomes between surgical patients with and without inflammatory skin disease.

Methods: We conducted a retrospective propensity score-matched cohort study using the TriNetX federated electronic health record network. Patients with inflammatory dermatoses undergoing surgery were matched 1:1 to controls without inflammatory skin disease. A secondary matched cohort of patients with postprocedural pain was analyzed to evaluate analgesic prescribing following documented postoperative pain. Outcomes included opioid and non-opioid analgesic use, postoperative pain, complications, and postoperative nausea and vomiting (PONV).

Results: After matching, the surgical cohort included 38,569 patients per group and the postprocedural-pain cohort included 149,002 patients per group. Thirty-day opioid use, the primary outcome, was lower in the inflammatory cohort than in controls (3.2% vs 7.0%; risk difference (RD), -3.8 percentage points; 95% confidence interval (CI), -4.2 to -3.4; risk ratio (RR), 0.46; 95% CI, 0.42-0.50), with lower opioid and non-opioid analgesic use persisting across the evaluated follow-up periods. Despite reduced analgesic use, recorded postoperative pain was similar at 30 days and higher during longer follow-up. PONV occurred more frequently in patients with inflammatory dermatoses, while most other postoperative complications were comparable between groups.

Conclusions: Patients with inflammatory dermatoses received less perioperative systemic analgesia despite no reduction in recorded postoperative pain. Because lower prescribing alone cannot establish undertreatment without validated pain severity or patient-reported outcomes, we interpret these associations as hypothesis-generating; they identify a potential difference in perioperative pain management and highlight the need for prospective studies to better define analgesic needs in surgical patients with inflammatory skin disease.

## Introduction

Inflammatory dermatoses, including atopic and contact dermatitis, psoriasis, hidradenitis suppurativa, bullous disorders, pyoderma gangrenosum, rosacea, and urticaria, are chronic immune-mediated conditions associated with sustained systemic inflammation. Several of these conditions are accompanied by altered sensory processing and nociception; the pathways underlying pruritus and pain in inflammatory skin disease overlap and may be sensitized by chronic inflammation [1-3]. Whether these alterations translate into different postoperative pain experiences or different perioperative analgesic management is not well characterized.

Outside the surgical setting, patients with inflammatory dermatoses such as psoriasis and hidradenitis suppurativa receive more opioids than unaffected individuals [4,5], and disparities in pain assessment and analgesic prescribing are well documented across patient populations [6]. Whether this greater opioid exposure persists in the perioperative period when acute surgical pain dominates has not been examined. Adequate postoperative analgesia is a basic quality-of-care expectation; if surgical patients with inflammatory skin disease are instead systematically offered less analgesia, a clinically important and remediable difference would exist [7]. At the same time, contemporary perioperative care emphasizes multimodal, opioid-sparing analgesia and opioid stewardship [7,8], so lower opioid use could also reflect appropriate, guideline-concordant prescribing; distinguishing appropriate opioid avoidance from undertreatment therefore requires attention to concurrent non-opioid analgesia and to pain and recovery outcomes. Recent procedure-specific and consensus guidance has further advanced multimodal, opioid-sparing pathways and structured opioid stewardship as standards for perioperative care [9-11].

To address this gap, we used the TriNetX global federated electronic health record (EHR) network to compare perioperative analgesic use, recorded postoperative pain, and surgical outcomes between 1:1 propensity-matched surgical cohorts with and without inflammatory dermatoses, and to characterize analgesic prescribing after a matched postprocedural pain diagnosis. All estimates are interpreted as associations, not causal effects. The primary objective concerned the surgical cohort, with 30-day opioid analgesic use as the principal outcome and the 30-day window as the primary perioperative period; the 90-, 180-, and 365-day windows were prespecified as secondary, longer-term outcomes that may also capture ongoing or chronic analgesic use. The secondary objective was to characterize analgesic and dermatologic prescribing after a matched postprocedural-pain diagnosis.

This work was previously presented as an e-poster at the Medical Dermatology Society 2026 Annual Meeting on March 26, 2026, in Denver, Colorado.

## Materials and methods

Study design and reporting

We conducted a retrospective cohort study within the TriNetX Research Network [12]. TriNetX returns only de-identified, aggregate counts from participating health care organizations (HCOs); analyses of such aggregate data are exempt from institutional review board review. Two complementary analyses were prespecified: a primary surgical cohort and a secondary postprocedural-pain cohort. This study was reported in accordance with the Strengthening the Reporting of Observational Studies in Epidemiology (STROBE) guideline [13]. A completed STROBE checklist is provided in the Appendix.

Data source

Cohorts were defined within the TriNetX Research Network using standardized diagnosis (ICD-10-CM), procedure (SNOMED, CPT), medication (VA drug class), and laboratory code concepts. For the surgical cohort, the query was run on the network with 107 HCOs queried and 106 responding; for the postprocedural-pain cohort, 109 HCOs were queried and 109 responded. All primary queries and analyses were performed on December 5, 2025. Medication concepts are drawn from EHR medication records (medication orders or prescriptions and, where available, administrations) mapped to VA drug classes; they represent documented prescribing or administration and do not distinguish these from dispensing or confirm actual patient use.

Cohort definitions

In the primary surgical analysis, the inflammatory cohort comprised patients with at least one surgical procedure (SNOMED 387713003, i.e., any surgical procedure) and an inflammatory dermatosis diagnosis recorded at least one day before that procedure: bullous disorders (ICD-10-CM L10-L14), pyoderma gangrenosum (L88), dermatitis and eczema (L20-L30), granulomatous disorders of skin (L92), rosacea (L71), urticaria (L50), papulosquamous disorders (L40-L45), and other follicular disorders (L73). An identical set of exclusion diagnoses was applied to both cohorts: other and unspecified polyneuropathies (G62), other mononeuropathies (G58), opioid-related disorders (F11), long-term opiate analgesic use (Z79.891), fibromyalgia (M79.7), skin infections (L00-L08), melanoma and other skin malignancies (C43-C44), other congenital malformations (Q80-Q89), pressure ulcer (L89), radiation-related skin disorders (L55-L59), and long-term systemic steroid use (Z79.52). The control cohort comprised surgical patients who had none of the inflammatory dermatoses; because the same exclusions were applied to both groups, the cohorts differed only by the presence or absence of inflammatory skin disease. The index date was the first surgical procedure (SNOMED 387713003) meeting these criteria.

In the secondary postprocedural-pain analysis, a separate cohort (a distinct population, not the same patients as the surgical cohort) was defined by a diagnosis of other acute postprocedural pain (G89.18) or other chronic postprocedural pain (G89.28), with or without inflammatory dermatoses, applying the same exclusion list; the pain diagnosis defined the index date. This analysis examined analgesic and dermatologic prescribing specifically among patients documented with postprocedural pain.

Covariates and propensity-score matching

In both analyses, TriNetX performed 1:1 nearest-neighbor propensity-score matching (PSM) without replacement on 29 prespecified covariates spanning demographics (age at index, sex, race, ethnicity), cardiometabolic and respiratory comorbidities, psychiatric and pain-related diagnoses, immune/inflammatory conditions, and body mass index (BMI) [14]. Balance was assessed by the standardized mean difference (SMD), with |SMD| <0.10 considered well balanced; all 29 covariates were well balanced after matching (all |SMD| <0.06) [15]. The 29 covariates comprised 14 demographic characteristics (age at index; sex (female, male, unknown); race (White, Black or African American, Asian, American Indian or Alaska Native, Native Hawaiian or Other Pacific Islander, other, unknown); ethnicity (Hispanic or Latino, not Hispanic or Latino, unknown)), 14 diagnostic categories (hypertensive diseases (I10-I1A); disorders of lipoprotein metabolism (E78); other and unspecified metabolic disorders (E88); other chronic obstructive pulmonary disease (J44); asthma (J45); chronic kidney disease (N18); persistent mood (affective) disorders (F34); mental and behavioral disorders due to psychoactive substance use (F10-F19); inflammatory polyarthropathies (M05-M14); systemic connective tissue disorders (M30-M36); disorders of gallbladder, biliary tract and pancreas (K80-K87); diseases of the blood and blood-forming organs and certain disorders involving the immune mechanism (D50-D89); diabetes mellitus (E08-E13); and other chronic pain (G89.29)), and body mass index. Achieving |SMD| <0.10 demonstrates balance on the measured covariates but does not establish exchangeability with respect to unmeasured factors; in particular, procedure type and surgical severity were not among the matched covariates and remain potential confounders.

Outcomes

Surgical-cohort outcomes were postoperative pain (G89.18, G89.28, R52), opioid analgesics (VA class CN101), a non-opioid analgesic composite (nonsteroidal anti-inflammatory and antirheumatic products (ATC M01A) together with acetaminophen and gabapentinoids), postprocedural complications (T81), infection following a procedure (T81.4), wound dehiscence (T81.3), emergency department (ED) visits (SNOMED/CPT emergency-visit concepts plus ICD-10-CM Z76.89), and postoperative nausea and vomiting (PONV; nausea and vomiting, ICD-10-CM R11). Postprocedural-pain-cohort outcomes were nonsteroidal anti-inflammatory analgesics, non-opioid analgesics, opioid antagonist analgesics (opioid agents with antagonist or partial-agonist activity that are used for analgesia, such as buprenorphine, nalbuphine, butorphanol, and pentazocine, including naloxone-containing combination analgesics; these were analyzed as an analgesic class because the agents are prescribed for pain), and opioid analgesics, plus targeted anti-inflammatory topical agents (VA class DE200), which served as a disease-directed positive control, and, for context, other topical dermatologicals (VA class DE900) and all dermatological agents (ATC class D). The specific medication classes and representative agents in each analgesic category are listed in Table 1. Surgical-cohort outcomes were assessed at 30 days (the primary perioperative window) and, as prespecified secondary longer-term outcomes, at 90, 180, and 365 days after the index date; postprocedural-pain-cohort outcomes were assessed at 30, 90, and 180 days after the pain diagnosis. Analgesic use in windows beyond 30 days may capture ongoing or chronic analgesic use not directly attributable to the index procedure and is interpreted accordingly.

**Table 1:** Medication classes and representative agents in each analgesic and dermatologic category Category membership is defined by the TriNetX drug-class concept used in the query (VA drug class or ATC class as indicated). Representative agents are illustrative examples of each class and are not exhaustive.

Category	Definition (drug-class concept)	Representative agents
Opioid analgesics	VA drug class CN101 (opioid analgesics)	morphine, oxycodone, hydrocodone, hydromorphone, fentanyl, codeine, tramadol, methadone
Non-opioid analgesic composite (surgical cohort)	NSAIDs/antirheumatic products (ATC M01A) plus acetaminophen and gabapentinoids	ibuprofen, naproxen, diclofenac, celecoxib, ketorolac, meloxicam; acetaminophen; gabapentin, pregabalin
Nonsteroidal anti-inflammatory analgesics	NSAIDs/antirheumatic products (ATC M01A)	ibuprofen, naproxen, diclofenac, celecoxib, ketorolac, meloxicam, indomethacin
Non-opioid analgesics	Non-opioid analgesic drug-class concept (acetaminophen, NSAIDs, and gabapentinoids as captured by the platform)	acetaminophen, ibuprofen, naproxen, gabapentin, pregabalin
Opioid antagonist analgesics	Opioid agents with antagonist or partial-agonist activity used for analgesia (platform opioid-antagonist drug-class concept)	buprenorphine, nalbuphine, butorphanol, pentazocine, naloxone-containing combination analgesics
Targeted anti-inflammatory topical agents (positive control)	VA drug class DE200	topical corticosteroids (e.g., triamcinolone, hydrocortisone, clobetasol) and topical calcineurin inhibitors (tacrolimus, pimecrolimus)
Other topical dermatologicals (context)	VA drug class DE900	miscellaneous topical dermatologic agents
All dermatological agents (context)	ATC class D (dermatologicals)	all dermatologic medications

Statistical analysis

For each outcome, TriNetX risk analysis estimated the risk (patients with the outcome divided by patients in the cohort) in each matched cohort, excluding patients with the outcome before the start of the time window, so analytic denominators vary by outcome and window. We report risk ratios (RRs) with 95% confidence intervals (CIs) as the primary effect measure. A P value less than .05 was considered statistically significant. Analyses used the TriNetX analytics platform. In addition to RRs, we report absolute risk differences (RDs) with 95% CIs for all outcomes. The primary analyses of both cohorts, comprising all reported outcomes and the covariate-balance assessments, were performed on the TriNetX network on December 5, 2025. Two additional sensitivity analyses were performed subsequently, in August 2026, on the then-current network: procedure-specific analyses in two representative operations spanning surgical intensity (excision of colon, SNOMED 23968004; wrist procedures, SNOMED 118707002) and disaggregation of the non-opioid composite into acetaminophen, nonsteroidal anti-inflammatory drugs, and gabapentinoids. Each used the identical inflammatory-dermatosis definition, exclusion set, and 1:1 propensity-score matching and the 30-day primary window; because the network accrues additional records over time, because the network accrued additional records between December 2025 and August 2026, each was matched independently on the larger current network, so their cohort sizes and analytic denominators differ from, and for some drug classes exceed, those of the primary analysis; both are reported as supportive analyses directionally consistent with the primary results. The relative frequencies of individual dermatoses within the inflammatory cohort are summarized descriptively in the Results as proportions of the cohort.

## Results

Cohort characteristics

In the surgical analysis, the inflammatory cohort included 38,599 patients and the control cohort 3,368,793 before matching; 1:1 PSM yielded 38,569 patients per arm (Figure 1). All 29 covariates were well balanced after matching (e.g., age 32.8 vs 32.9 years; all |SMD| <0.06) (Table 2). In the separate postprocedural-pain analysis, the inflammatory cohort included 149,064 patients and the control cohort 1,220,578 before matching, yielding 149,002 patients per arm after propensity-score matching for the 30- and 180-day windows, with all 29 covariates well balanced (Table 3). TriNetX re-matched the cohorts for each time window, yielding 147,845 patients per arm for the 90-day analysis. The most common inflammatory dermatoses in the surgical inflammatory cohort were dermatitis and eczema (64%; n=30,721) and urticaria (29%; n=14,012), followed by papulosquamous disorders including psoriasis (7%; n=3,513), granulomatous disorders (5%; n=2,270), other follicular disorders including hidradenitis suppurativa (4%; n=1,924), bullous disorders (2%; n=944), rosacea (1%; n=716), and pyoderma gangrenosum (<1%, n=64). Patients could carry more than one qualifying dermatosis; therefore, these categories were not mutually exclusive and the percentages sum to more than 100%.

**Figure 1:**
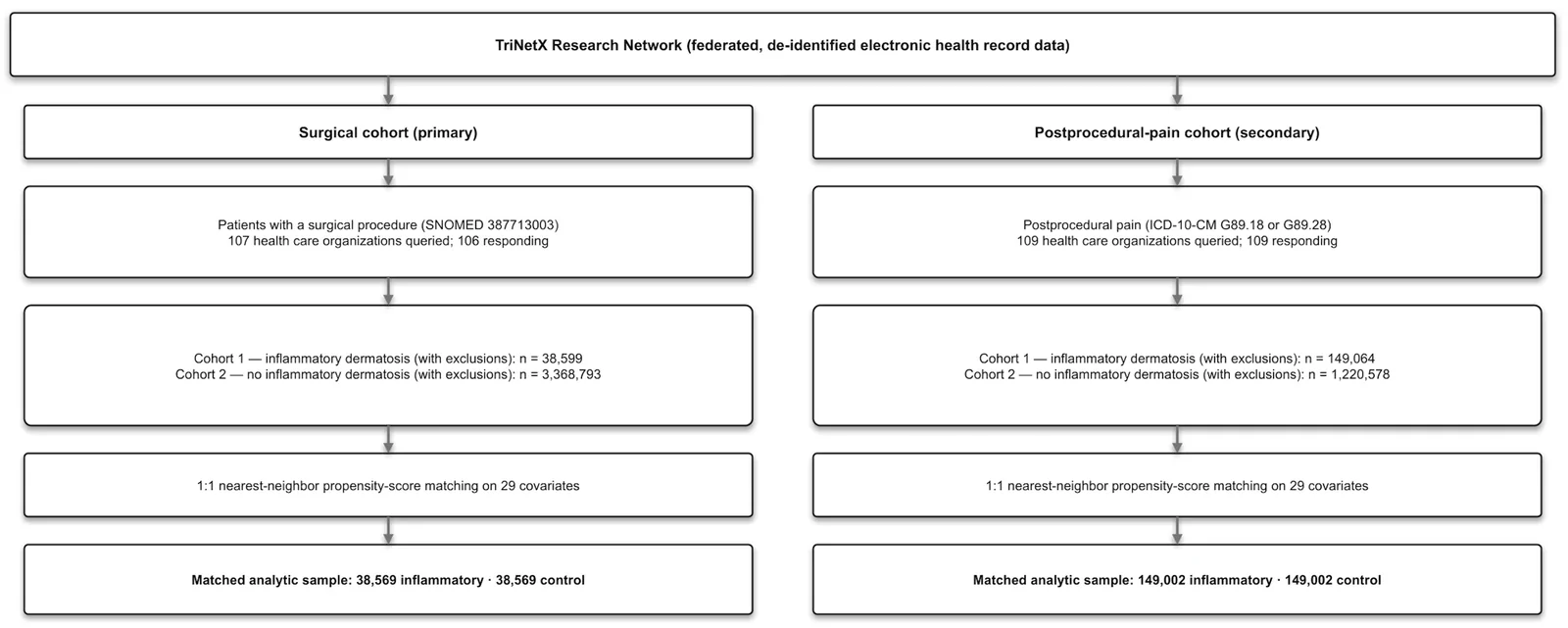
Cohort assembly and propensity-score matching. Derivation of the surgical (primary) and postprocedural-pain (secondary) cohorts from the TriNetX federated network. Each inflammatory-dermatosis cohort was compared with a non-dermatosis cohort under identical prespecified exclusions and matched 1:1 on 29 covariates, yielding 38,569 (surgical) and 149,002 (postprocedural-pain) matched pairs. Prespecified exclusions, applied identically to both groups, were neuropathies (polyneuropathy, mononeuropathy), opioid-related disorders and long-term opioid use, fibromyalgia, skin infection, skin cancer, congenital malformations, pressure ulcer, radiation-related skin disorders, and long-term systemic corticosteroid use. HCO indicates health care organization.

**Table 2:** Surgical-cohort post-match covariate balance. Cohorts were 1:1 propensity-score matched using nearest-neighbor matching on 29 covariates within TriNetX. A SMD <0.10 indicates adequate balance. Values are percentages of the cohort unless otherwise indicated. All 29 covariates were well balanced after matching (all |SMD| <0.06). Abbreviations: BMI: Body mass index; SMD: Standardized mean difference.

Characteristic	Inflammatory (n=38,569)	Control (n=38,569)	SMD
Age at index, mean (SD), y	32.8 (24.6)	32.9 (24.6)	0.005
Female	52.3%	52.4%	<0.001
Male	47.6%	47.6%	<0.001
Unknown sex	0.0%	0.0%	NA
White	28.4%	28.7%	0.005
Black or African American	5.3%	5.1%	0.009
Asian	0.6%	0.6%	0.006
American Indian or Alaska Native	0.1%	0.1%	0.006
Native Hawaiian or Other Pacific Islander	0.1%	0.1%	0.004
Other Race	12.6%	12.9%	0.010
Unknown Race	53.0%	52.6%	0.009
Hispanic or Latino	2.7%	2.9%	0.007
Not Hispanic or Latino	17.6%	17.7%	0.002
Unknown Ethnicity	79.6%	79.4%	0.005
Hypertensive diseases	10.6%	11.3%	0.023
Disorders of lipoprotein metabolism and other lipidemias	2.9%	2.9%	0.005
Other and unspecified metabolic disorders	0.9%	0.8%	0.009
Other chronic obstructive pulmonary disease	0.8%	0.7%	0.008
Asthma	8.8%	8.5%	0.009
Chronic kidney disease (CKD)	1.1%	1.1%	0.008
Persistent mood [affective] disorders	0.3%	0.2%	0.017
Mental and behavioral disorders due to psychoactive substance use	0.5%	0.4%	0.018
Inflammatory polyarthropathies	1.7%	1.7%	0.007
Systemic connective tissue disorders	1.0%	0.9%	0.015
Disorders of gallbladder, biliary tract and pancreas	3.1%	2.9%	0.007
Diseases of the blood and blood-forming organs and certain disorders involving the immune mechanism	4.4%	4.2%	0.011
Diabetes mellitus	3.0%	3.1%	0.005
Other chronic pain	1.0%	0.8%	0.021
BMI, mean (SD), kg/m²	22.8 (7.0)	23.1 (7.4)	0.053

**Table 3:** Postprocedural-pain-cohort post-match covariate balance Cohorts were 1:1 propensity-score matched on 29 covariates within TriNetX. A standardized mean difference (SMD) <0.10 indicates adequate balance; all covariates were well balanced after matching (all |SMD| <0.06). Values are percentages of the cohort unless otherwise indicated. Abbreviations: SMD, standardized mean difference.

Characteristic	Inflammatory (n=149,002)	Control (n=149,002)	SMD
Age at index, mean (SD), y	45.3 (22.1)	45.6 (21.9)	0.010
Female	64.9%	64.7%	0.003
Male	35.1%	35.3%	0.003
Unknown sex	0.0%	0.0%	NA
White	67.9%	69.3%	0.029
Black or African American	18.2%	18.0%	0.006
Asian	3.4%	3.1%	0.017
American Indian or Alaska Native	0.5%	0.5%	0.011
Native Hawaiian or Other Pacific Islander	0.6%	0.6%	0.009
Other Race	4.8%	4.5%	0.013
Unknown Race	4.5%	4.1%	0.020
Hispanic or Latino	9.2%	8.8%	0.014
Not Hispanic or Latino	77.1%	77.8%	0.018
Unknown Ethnicity	13.7%	13.3%	0.010
Hypertensive diseases	40.9%	41.8%	0.019
Disorders of lipoprotein metabolism and other lipidemias	38.2%	39.2%	0.021
Other and unspecified metabolic disorders	3.4%	2.9%	0.028
Other chronic obstructive pulmonary disease	5.9%	5.6%	0.012
Asthma	20.8%	20.9%	0.001
Chronic kidney disease (CKD)	7.5%	7.2%	0.012
Persistent mood [affective] disorders	2.6%	2.3%	0.022
Mental and behavioral disorders due to psychoactive substance use	19.7%	19.8%	0.004
Inflammatory polyarthropathies	11.2%	10.8%	0.012
Systemic connective tissue disorders	2.7%	2.4%	0.019
Disorders of gallbladder, biliary tract and pancreas	11.6%	11.3%	0.010
Diseases of the blood and blood-forming organs and certain disorders involving the immune mechanism	35.6%	35.9%	0.005
Diabetes mellitus	15.9%	15.6%	0.008
Other chronic pain	29.3%	29.2%	0.002
BMI, mean (SD), kg/m²	29.7 (8.0)	29.9 (8.1)	0.015

Surgical cohort: Systemic analgesia

In the surgical cohort, the inflammatory group received less systemic analgesia at every window. Opioid use was lower at 30 days (633/19,647 (3.2%) vs 1,542/22,017 (7.0%); RR, 0.46 [0.42-0.50]; P<0.001), 90 days (4.7% vs 8.2%; RR, 0.57 [0.53-0.62]), 180 days (6.1% vs 9.3%; RR, 0.65 [0.61-0.70]), and 365 days (8.5% vs 11.1%; RR, 0.77 [0.72-0.81]; all P<0.001). The non-opioid composite was likewise lower at 30 days (4.8% vs 8.2%; RR, 0.59 [0.54-0.64]), 90 days (7.3% vs 9.8%; RR, 0.74 [0.69-0.80]), and 180 days (9.7% vs 11.6%; RR, 0.84 [0.79-0.90]; all P<0.001), converging by 365 days (13.4% vs 13.9%; RR, 0.96 [0.91-1.02]; P=0.21). The 30-day opioid risk difference was -3.8 percentage points (95% CI, -4.2 to -3.4), corresponding to approximately 38 fewer patients per 1,000 receiving an opioid (Table 4). Disaggregating the non-opioid composite at 30 days, all three components were lower in the inflammatory cohort, most markedly NSAIDs (RR, 0.64 [0.59-0.69]), followed by gabapentinoids (RR, 0.77 [0.64-0.92]) and acetaminophen (RR, 0.79 [0.74-0.85]) (Table 5).

**Table 4:** Outcomes by inflammatory-dermatosis status in the surgical and postprocedural-pain cohorts Both cohorts were 1:1 propensity-score matched (surgical, 38,569 per arm; postprocedural-pain, 149,002 per arm for the 30- and 180-day windows and 147,845 per arm for the 90-day window, which was queried separately). Risk = patients with the outcome divided by patients in the cohort within each window; analytic denominators (N) vary by outcome and window because patients with the outcome before the window are excluded. Risk difference (RD) and risk ratio (RR) are inflammatory vs control and are shown for each outcome and window. The surgical non-opioid analgesic composite comprises nonsteroidal anti-inflammatory/antirheumatic products, acetaminophen, and gabapentinoids. In the postprocedural-pain cohort, targeted anti-inflammatory topical agents served as a disease-directed positive control; other topical dermatologicals and all dermatological agents are broad categories shown for context. Abbreviations: CI: Confidence interval; RD: Risk difference; RR: Risk ratio.

Outcome or medication class	Window (d)	Inflammatory % (n/N)	Control % (n/N)	Risk difference (95% CI)	Risk ratio (95% CI)	P value
Surgical cohort (38,569 per arm)						
Postoperative pain	30	0.41% (144/34,964)	0.40% (151/37,486)	0.000 (-0.001 to 0.001)	1.02 (0.81-1.28)	0.85
	90	0.78% (273/34,964)	0.63% (238/37,486)	0.001 (0.000 to 0.003)	1.23 (1.03-1.46)	0.02
	180	1.22% (428/34,964)	0.84% (315/37,486)	0.004 (0.002 to 0.005)	1.46 (1.26-1.68)	<0.001
	365	1.90% (664/34,964)	1.21% (452/37,486)	0.007 (0.005 to 0.009)	1.57 (1.40-1.77)	<0.001
Opioid analgesics	30	3.22% (633/19,647)	7.00% (1,542/22,017)	-0.038 (-0.042 to -0.034)	0.46 (0.42-0.50)	<0.001
	90	4.69% (921/19,647)	8.23% (1,812/22,017)	-0.035 (-0.040 to -0.031)	0.57 (0.53-0.62)	<0.001
	180	6.10% (1,199/19,647)	9.34% (2,056/22,017)	-0.032 (-0.037 to -0.027)	0.65 (0.61-0.70)	<0.001
	365	8.49% (1,668/19,647)	11.06% (2,436/22,017)	-0.026 (-0.031 to -0.020)	0.77 (0.72-0.81)	<0.001
Non-opioid analgesic composite (NSAIDs, acetaminophen, gabapentinoids)	30	4.78% (606/12,669)	8.16% (1,567/19,197)	-0.034 (-0.039 to -0.028)	0.59 (0.54-0.64)	<0.001
	90	7.29% (924/12,669)	9.85% (1,890/19,197)	-0.026 (-0.032 to -0.019)	0.74 (0.69-0.80)	<0.001
	180	9.72% (1,232/12,669)	11.55% (2,218/19,197)	-0.018 (-0.025 to -0.011)	0.84 (0.79-0.90)	<0.001
	365	13.43% (1,702/12,669)	13.93% (2,674/19,197)	-0.005 (-0.013 to 0.003)	0.96 (0.91-1.02)	0.21
Postprocedural complications	30	0.19% (74/38,358)	0.17% (65/38,494)	0.000 (0.000 to 0.001)	1.14 (0.82-1.59)	0.43
	90	0.26% (99/38,358)	0.23% (87/38,494)	0.000 (0.000 to 0.001)	1.14 (0.86-1.52)	0.36
	180	0.33% (126/38,358)	0.27% (103/38,494)	0.001 (0.000 to 0.001)	1.23 (0.95-1.59)	0.12
	365	0.41% (156/38,358)	0.31% (121/38,494)	0.001 (0.000 to 0.002)	1.29 (1.02-1.64)	0.03
Infection following a procedure	30	0.03% (10/38,529)	0.04% (15/38,557)	0.000 (0.000 to 0.000)	0.67 (0.30-1.49)	0.32
	90	0.06% (22/38,529)	0.05% (20/38,557)	0.000 (0.000 to 0.000)	1.10 (0.60-2.02)	0.76
	180	0.07% (27/38,529)	0.05% (20/38,557)	0.000 (0.000 to 0.001)	1.35 (0.76-2.41)	0.31
	365	0.09% (34/38,529)	0.07% (26/38,557)	0.000 (0.000 to 0.001)	1.31 (0.79-2.18)	0.30
Wound dehiscence	30	0.06% (25/38,515)	0.05% (21/38,551)	0.000 (0.000 to 0.000)	1.19 (0.67-2.13)	0.55
	90	0.09% (36/38,515)	0.08% (32/38,551)	0.000 (0.000 to 0.001)	1.13 (0.70-1.81)	0.62
	180	0.11% (42/38,515)	0.10% (39/38,551)	0.000 (0.000 to 0.001)	1.08 (0.70-1.67)	0.74
	365	0.12% (47/38,515)	0.12% (45/38,551)	0.000 (0.000 to 0.001)	1.05 (0.70-1.57)	0.83
Emergency department visits	30	0.32% (110/33,898)	0.29% (105/35,759)	0.000 (-0.001 to 0.001)	1.11 (0.85-1.44)	0.46
	90	0.62% (210/33,898)	0.55% (195/35,759)	0.001 (0.000 to 0.002)	1.14 (0.94-1.38)	0.20
	180	0.97% (330/33,898)	0.91% (326/35,759)	0.001 (-0.001 to 0.002)	1.07 (0.92-1.24)	0.40
	365	1.56% (529/33,898)	1.47% (525/35,759)	0.001 (-0.001 to 0.003)	1.06 (0.94-1.20)	0.32
Postoperative nausea and vomiting	30	0.35% (114/32,881)	0.19% (69/36,465)	0.002 (0.001 to 0.002)	1.83 (1.36-2.47)	<0.001
	90	0.82% (269/32,881)	0.42% (153/36,465)	0.004 (0.003 to 0.005)	1.95 (1.60-2.38)	<0.001
	180	1.49% (489/32,881)	0.71% (260/36,465)	0.008 (0.006 to 0.009)	2.09 (1.80-2.42)	<0.001
	365	2.55% (837/32,881)	1.25% (454/36,465)	0.013 (0.011 to 0.015)	2.04 (1.83-2.29)	<0.001
Postprocedural-pain cohort (149,002 per arm; 147,845 for the 90-day window)						
Nonsteroidal anti-inflammatory analgesics	30	8.55% (6,586/77,063)	9.17% (8,535/93,104)	-0.006 (-0.009 to -0.004)	0.93 (0.90-0.96)	<0.001
	90	10.07% (7,680/76,290)	10.56% (9,698/91,868)	-0.005 (-0.008 to -0.002)	0.95 (0.93-0.98)	0.001
	180	11.43% (8,806/77,063)	11.78% (10,971/93,104)	-0.004 (-0.007 to -0.001)	0.97 (0.95-1.00)	0.02
Non-opioid analgesics	30	26.66% (3,415/12,808)	33.92% (5,313/15,665)	-0.073 (-0.083 to -0.062)	0.79 (0.76-0.82)	<0.001
	90	30.79% (3,901/12,669)	38.10% (6,030/15,826)	-0.073 (-0.084 to -0.062)	0.81 (0.78-0.84)	< 0.001
	180	33.28% (4,262/12,808)	40.17% (6,293/15,665)	-0.069 (-0.080 to -0.058)	0.83 (0.80-0.85)	<0.001
Opioid antagonist analgesics	30	4.89% (4,489/91,794)	5.66% (5,282/93,393)	-0.008 (-0.010 to -0.006)	0.86 (0.83-0.90)	<0.001
	90	5.67% (5,189/91,449)	6.52% (6,092/93,454)	-0.008 (-0.011 to -0.006)	0.87 (0.84-0.90)	<0.001
	180	6.49% (5,962/91,794)	7.33% (6,842/93,393)	-0.008 (-0.011 to -0.006)	0.89 (0.86-0.92)	<0.001
Opioid analgesics	30	30.75% (3,494/11,361)	33.95% (4,378/12,894)	-0.032 (-0.044 to -0.020)	0.91 (0.87-0.94)	<0.001
	90	35.51% (4,001/11,267)	38.49% (4,986/12,955)	-0.030 (-0.042 to -0.018)	0.92 (0.89-0.95)	<0.001
	180	37.56% (4,267/11,361)	40.51% (5,223/12,894)	-0.029 (-0.042 to -0.017)	0.93 (0.90-0.96)	<0.001
Other topical dermatologicals	30	14.28% (2,369/16,590)	19.85% (3,777/19,026)	-0.056 (-0.063 to -0.048)	0.72 (0.69-0.75)	<0.001
	90	17.94% (2,950/16,444)	23.04% (4,363/18,938)	-0.051 (-0.059 to -0.043)	0.78 (0.75-0.81)	<0.001
	180	20.68% (3,430/16,590)	25.65% (4,881/19,026)	-0.050 (-0.059 to -0.041)	0.81 (0.78-0.84)	<0.001
All dermatological agents	30	15.22% (720/4,732)	26.06% (2,532/9,717)	-0.108 (-0.122 to -0.095)	0.58 (0.54-0.63)	<0.001
	90	20.54% (957/4,660)	30.95% (2,967/9,585)	-0.104 (-0.119 to -0.089)	0.66 (0.62-0.71)	<0.001
	180	25.02% (1,184/4,732)	34.39% (3,342/9,717)	-0.094 (-0.109 to -0.078)	0.73 (0.69-0.77)	<0.001
Targeted anti-inflammatory topical agents (positive control)	30	5.59% (2,671/47,756)	5.07% (4,573/90,267)	0.005 (0.003 to 0.008)	1.10 (1.05-1.16)	<0.001
	90	8.56% (4,038/47,186)	7.36% (6,551/89,035)	0.012 (0.009 to 0.015)	1.16 (1.12-1.21)	<0.001
	180	11.79% (5,632/47,756)	9.79% (8,835/90,267)	0.020 (0.017 to 0.024)	1.20 (1.17-1.24)	<0.001

**Table 5:** Non-opioid analgesic composite disaggregated by component (surgical cohort, 30-day window) Disaggregation of the non-opioid analgesic composite in the all-surgery surgical cohort at the 30-day window, performed as an August 2026 sensitivity analysis with independent 1:1 propensity-score matching (see Methods). The opioid-analgesics row is included only as a within-analysis reference; its risk ratio (0.48) therefore differs slightly from the primary December 2025 estimate (0.46) in Table 4. The composite reduction reflects lower acetaminophen and NSAID use, with gabapentinoid use also lower. Risk ratio is inflammatory versus control.

Component	Inflammatory % (n/N)	Control % (n/N)	Risk ratio (95% CI)	P value
Acetaminophen	4.36% (1,220/27,987)	5.49% (1,763/32,104)	0.79 (0.74-0.85)	<0.001
NSAIDs	4.58% (847/18,486)	7.18% (1,897/26,425)	0.64 (0.59-0.69)	<0.001
Gabapentinoids	0.44% (203/45,768)	0.58% (267/46,309)	0.77 (0.64-0.92)	0.005
Opioid analgesics (reference)	3.68% (809/21,979)	7.72% (1,884/24,391)	0.48 (0.44-0.52)	<0.001

Surgical cohort: Recorded postoperative pain

Recorded postoperative pain was similar at 30 days (144/34,964 (0.41%) vs 151/37,486 (0.40%); RR, 1.02 [0.81-1.28]; P=0.85) and modestly higher in the inflammatory cohort over longer follow-up (90 days, 0.78% vs 0.63%; RR, 1.23 [1.03-1.46]; P=.02; 180 days, 1.22% vs 0.84%; RR, 1.46 [1.26-1.68]; P<.001; 365 days, 1.90% vs 1.21%; RR, 1.57 [1.40-1.77]; P<0.001) (Table 4; Figure 2). The reduced analgesia was thus not accompanied by lower recorded pain.

**Figure 2:**
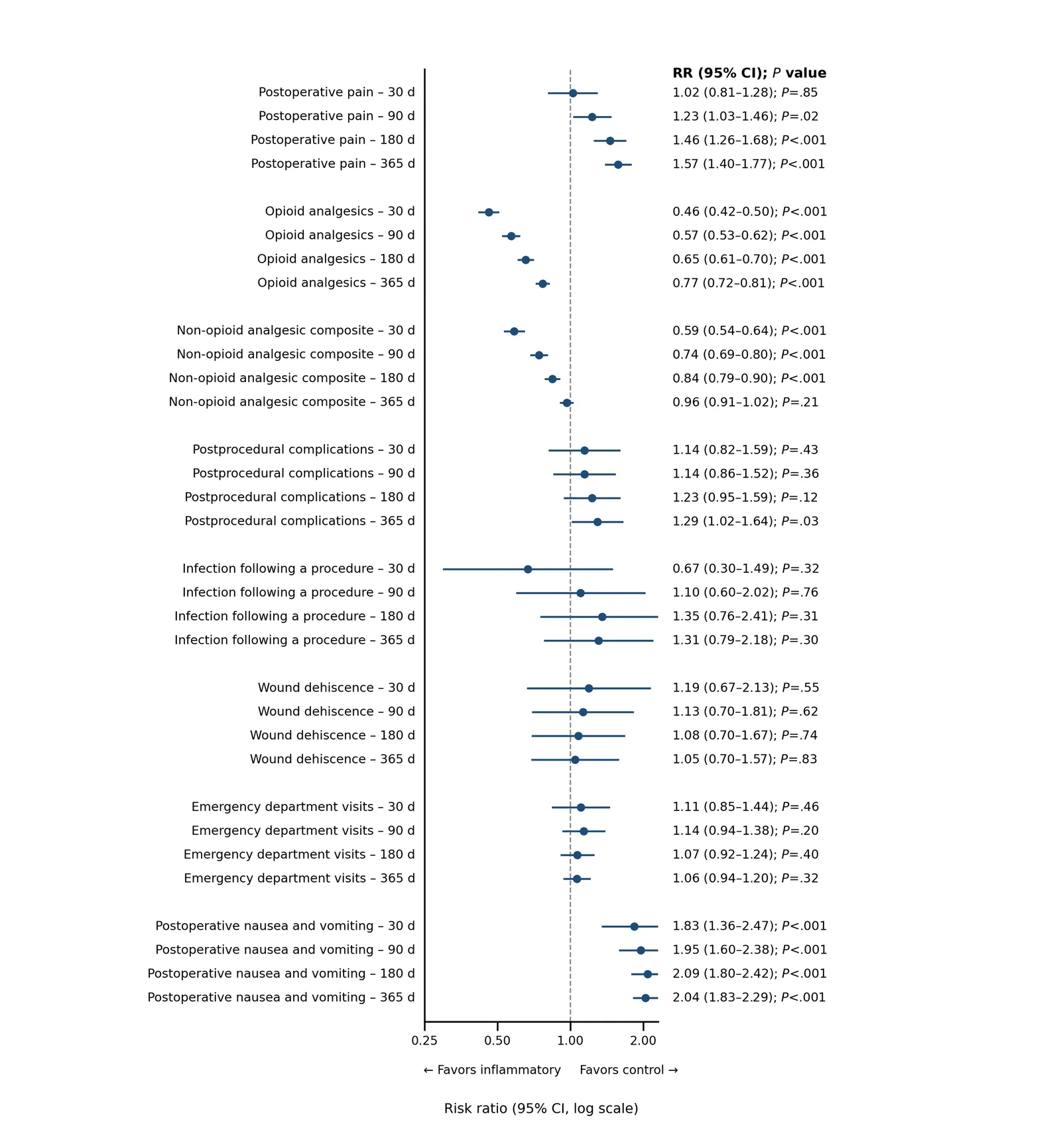
Forest plot of risk ratios with 95% confidence intervals (CIs) for surgical-cohort outcomes comparing the inflammatory and control cohorts. Risk ratios <1 favor the inflammatory cohort (lower risk); the dashed reference line marks a risk ratio of 1.0 on a log scale. All eight surgical-cohort outcomes are shown at the 30-, 90-, 180-, and 365-day windows.

Surgical cohort: Complications and PONV

Postprocedural complications, infection, wound dehiscence, and ED visits were low and largely similar between groups (e.g., 30-day complications RR, 1.14 [0.82-1.59]; P=0.43; infection RR, 0.67 [0.30-1.49]; P=0.32; wound dehiscence RR, 1.19 [0.67-2.13]; P=.55; ED visits RR, 1.11 [0.85-1.44]; P=.46), with a modest excess of complications in the inflammatory cohort by 365 days (RR, 1.29 [1.02-1.64]; P=0.03). PONV was consistently higher in the inflammatory cohort (30 days, 0.35% vs 0.19%; RR, 1.83 [1.36-2.47]; 90 days, RR, 1.95 [1.60-2.38]; 180 days, 1.49% vs 0.71%; RR, 2.09 [1.80-2.42]; 365 days, 2.55% vs 1.25%; RR, 2.04 [1.83-2.29]; all P<0.001) (Table 4). Because opioids are a leading cause of PONV, higher PONV despite lower opioid use indicates that the reduced analgesia is unlikely to reflect a milder or less painful surgical course in the inflammatory cohort [16,17]. Risk ratios for the surgical-cohort outcomes across follow-up intervals are summarized in Figure 2. PONV risk factors such as anesthetic technique, specific procedure type, and prior PONV were not captured; age and sex were balanced by matching. Because PONV was identified with the nonspecific nausea and vomiting code (R11), which does not by itself establish a postoperative or causal relationship to the index procedure, this outcome is most interpretable within the 30-day window. Full absolute risk differences with 95% CIs for all outcomes are provided in Table 4.

In procedure-specific sensitivity analyses at the 30-day primary window, opioid and non-opioid (NSAID) analgesic use were both lower in the inflammatory cohort within a major abdominal (excision of colon: opioid RR, 0.43 [0.26-0.70]; NSAID RR, 0.52 [0.33-0.80]) and a minor orthopedic (wrist: opioid RR, 0.74 [0.61-0.90]; NSAID RR, 0.66 [0.53-0.83]) operation, mirroring the all-surgery findings. Less common outcomes had too few events within a single procedure at 30 days to estimate reliably (for example, wrist PONV comprised 11 and 13 events, and colon-excision postoperative pain and PONV fell below reportable thresholds); aggregating across procedures in the all-surgery cohort provides the power to characterize the full perioperative-outcome spectrum (Table 6).

**Table 6:** Procedure-specific sensitivity analyses at the 30-day primary window (excision of colon; wrist procedures) Procedure-specific sensitivity analyses at the 30-day window in two representative operations (excision of colon, SNOMED 23968004; wrist procedures, SNOMED 118707002), performed as an August 2026 sensitivity analysis using the identical inflammatory-dermatosis definition, exclusion set, and 1:1 propensity-score matching (see Methods). Outcomes with event counts below the platform reportable threshold within a procedure are not shown (colon-excision postoperative pain and PONV). Risk ratio is inflammatory versus control. NSAID: Nonsteroidal anti-inflammatory drug; PONV: Postoperative nausea and vomiting.

Procedure	Outcome	Inflammatory % (n/N)	Control % (n/N)	Risk ratio (95% CI)	P value
Excision of colon	Opioid analgesics	2.44% (20/820)	5.72% (70/1,224)	0.43 (0.26-0.70)	<0.001
	NSAID analgesics	2.28% (26/1,141)	4.42% (78/1,765)	0.52 (0.33-0.80)	0.002
Wrist procedure	Postoperative pain	0.21% (14/6,712)	0.35% (25/7,168)	0.60 (0.31-1.15)	0.12
	Opioid analgesics	3.74% (166/4,440)	5.03% (254/5,046)	0.74 (0.61-0.90)	0.002
	NSAID analgesics	4.79% (107/2,235)	7.20% (263/3,651)	0.66 (0.53-0.83)	<0.001
	PONV	0.17% (11/6,410)	0.18% (13/7,055)	0.93 (0.42-2.08)	0.86

Postprocedural-pain cohort: Medication use

In the separately matched postprocedural-pain cohort, a distinct population of patients documented with postprocedural pain, the inflammatory group again received less systemic analgesia across every class. At 30 days, use was lower for non-opioid analgesics (26.7% vs 33.9%; RR, 0.79 [0.76-0.82]), opioid analgesics (30.8% vs 34.0%; RR, 0.91 [0.87-0.94]), opioid antagonist analgesics (4.9% vs 5.7%; RR, 0.86 [0.83-0.90]), and nonsteroidal anti-inflammatory analgesics (8.5% vs 9.2%; RR, 0.93 [0.90-0.96]; all P<.001), and differences persisted through 180 days (e.g., non-opioid analgesics 33.3% vs 40.2%; RR, 0.83 [0.80-0.85]; P<.001). In contrast, use of targeted anti-inflammatory topical agents, a disease-directed positive control, was higher in the inflammatory cohort and increased over time (30 days, 5.6% vs 5.1%; RR, 1.10 [1.05-1.16]; 180 days, 11.8% vs 9.8%; RR, 1.20 [1.17-1.24]; all P<.001), consistent with recognition and treatment of active skin disease. This rising topical use may also reflect misattribution of postprocedural pain to the underlying dermatosis, prompting disease-directed topical therapy in place of systemic analgesia. Broader dermatologic categories (other topical dermatologicals and all dermatological agents) were lower in the inflammatory cohort (30-day RR, 0.72 [0.69-0.75] and 0.58 [0.54-0.63]); because these categories overlap with and include the targeted agents, they are reported for context (Table 4; Figure 3).

**Figure 3:**
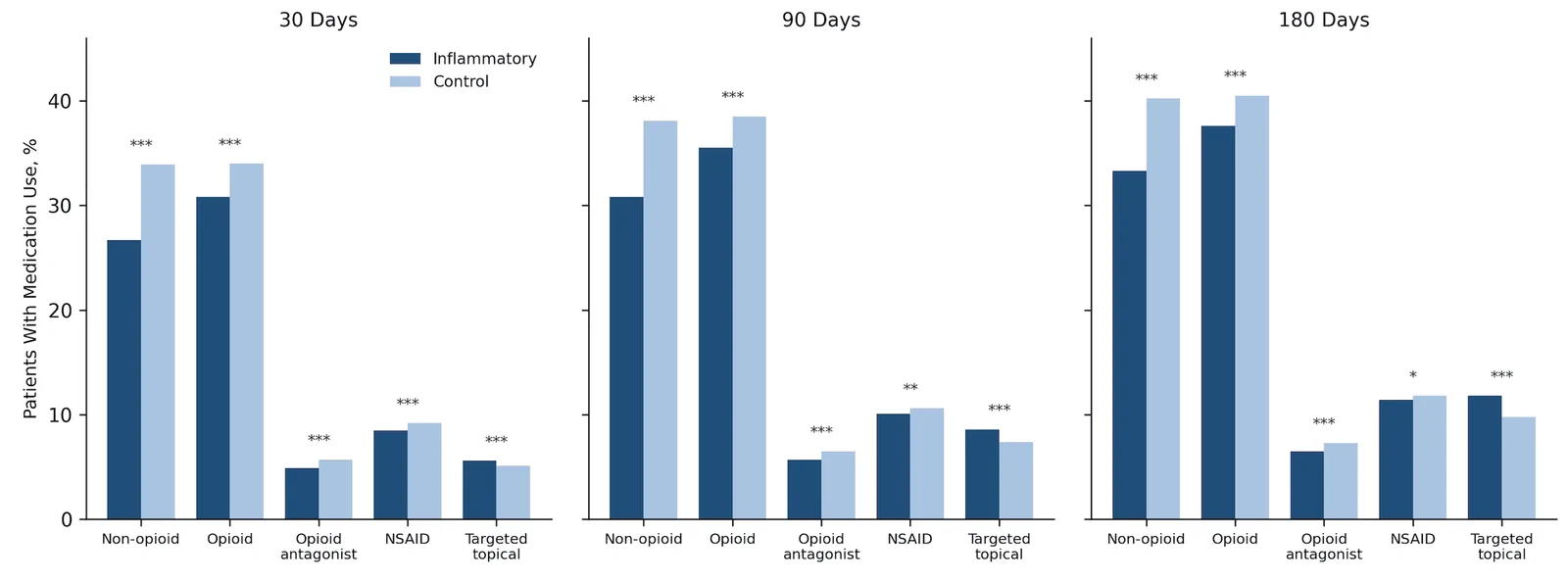
Medication use in the postprocedural-pain cohort by inflammatory-dermatosis status. Bars show the percentage of patients in the inflammatory-dermatosis group and the matched control group who received each medication class within 30, 90, and 180 days after the postprocedural-pain diagnosis. Non-opioid analgesics, opioid analgesics, opioid antagonist analgesics, and nonsteroidal anti-inflammatory (NSAID) analgesics are systemic classes; targeted anti-inflammatory topical agents are a disease-directed positive control that validates active skin disease in the inflammatory cohort. Asterisks denote the significance of the inflammatory vs control comparison (risk ratio) for each class and window: *P<.05, **P<.01, ***P<.001; NS, not significant.

## Discussion

In two large propensity-matched cohorts drawn from a global federated EHR network, surgical patients with inflammatory skin disease received markedly less systemic analgesia across both opioid and non-opioid classes than matched controls. Because the two analyses drew on separate, independently matched populations, the same reduction appearing in a cohort defined by a documented postprocedural pain diagnosis indicates the divergence is not explained simply by who is coded as having pain. This pattern runs counter to the established nonsurgical literature, in which patients with inflammatory dermatoses receive more opioids than unaffected individuals [4,5], and identifies the perioperative period as a setting where that relationship reverses. The reduced analgesia was not accompanied by lower recorded postoperative pain, which was similar at 30 days and higher over longer follow-up, so the analgesia difference does not track lower pain.

Several findings argue against the observed difference being a mere coding artifact. Targeted anti-inflammatory topical use, a disease-directed positive control, was higher in the inflammatory cohort and rose over time, showing that clinicians recognized and treated the underlying skin disease even as they prescribed less systemic analgesia; this trend may also indicate that postsurgical pain was sometimes attributed to the dermatosis and treated topically rather than with systemic analgesics. In addition, PONV was roughly twofold higher in the inflammatory cohort at every window despite lower opioid use, a pattern that argues against a uniformly milder surgical course and instead points to a genuinely different perioperative experience [8,17,18].

A biological contribution is plausible: chronic cutaneous inflammation is associated with altered nociceptive and pruriceptive signaling [1-3], which could change both how pain is experienced and how it is communicated and interpreted. However, the magnitude and consistency of the analgesia reduction, combined with the potential for residual confounding, mean that clinician decision-making, differential care-seeking, and unmeasured confounding remain equally compatible with the data. We therefore frame these results as a hypothesis-generating signal of a potential perioperative pain-management difference. Lower opioid use could also reflect appropriate, guideline-concordant opioid avoidance or stewardship rather than undertreatment; however, the parallel reduction in non-opioid analgesia and the higher PONV argue against selective, appropriate opioid-sparing as the sole explanation. Lower prescribing alone does not establish undertreatment: without validated pain severity, functional status, or patient-reported outcomes, the observed prescribing differences cannot be equated with inadequate analgesia.

This study has important limitations. It is observational and based on aggregate de-identified EHR data, so all estimates are associations rather than causal effects, and residual confounding cannot be excluded [19]. The exposure spans a heterogeneous group of dermatoses, and the cohort was defined by any surgical procedure (SNOMED 387713003) rather than a specific procedure type, so the findings are not procedure-specific; because procedure type and surgical severity were not matched, differences in the mix of procedures between groups could contribute to the observed analgesic differences. Medications obtained over the counter or outside contributing health care organizations are also incompletely captured [19]. Several additional limitations bear on interpretation. The inflammatory dermatoses were analyzed collectively as a single exposure rather than individually, and the distribution of specific conditions is summarized in the Results, but per-condition effect estimates were not derived; the group is clinically heterogeneous. The non-opioid analgesic composite combines nonsteroidal anti-inflammatory drugs, acetaminophen, and gabapentinoids, which differ in indication and mechanism; these were disaggregated at the 30-day primary window (Table 5). Procedure type and surgical severity were neither matched nor available for stratification, so differences in procedure mix could contribute to the findings, and procedure-specific sensitivity analyses in representative operations showed directionally consistent associations, although single-procedure cohorts are underpowered for less common outcomes, which is why the aggregated all-surgery cohort was used for the primary analysis. PONV was ascertained with a nonspecific nausea and vomiting code that does not confirm a postoperative etiology or temporal link to surgery, particularly at the longer windows. Validated pain severity, functional status, patient-reported outcomes, and analgesic dose and duration were not available, so lower utilization cannot be equated with undertreatment. Finally, balance on measured covariates does not ensure exchangeability with respect to unmeasured factors.

## Conclusions

In matched cohorts from a global federated EHR network, surgical patients with inflammatory skin disease received less systemic analgesia across opioid and non-opioid classes than matched controls, while recorded postoperative pain was not lower and postoperative nausea and vomiting was more frequent. The direction of this perioperative pattern contrasts with the greater opioid exposure previously described in inflammatory dermatoses outside surgical settings. These findings are associations rather than causal effects and identify a potentially remediable perioperative analgesia difference, although residual confounding could account for part of the signal and lower prescribing alone does not establish undertreatment. Prospective, diagnosis- and procedure-specific studies that incorporate validated pain severity, analgesic dose and duration, and patient-reported outcomes are needed to determine whether these prescribing differences represent clinically meaningful differences in pain management and to clarify the mechanisms underlying them.

## Appendices

**Table 7:** STROBE statement, checklist of items for cohort studies The Location column cites the relevant manuscript section, table, or figure.

Item	No.	Description / how addressed	Location
Title and abstract	1	Cohort design indicated in title; structured abstract summarizes design, cohorts, outcomes, and results.	Title; Abstract
Background/rationale	2	Inflammatory dermatoses, altered nociception, and the unknown perioperative pain-management pattern.	Introduction
Objectives	3	Compare recorded postoperative pain, analgesic use, and surgical outcomes by inflammatory-dermatosis status.	Introduction (final paragraph)
Study design	4	Retrospective propensity-matched cohort study in TriNetX.	Methods: Study Design
Setting	5	Global federated EHR network; 107 HCOs queried; queries run Dec 5, 2025.	Methods: Data Source
Participants	6	Surgical and postprocedural-pain cohorts with inclusion/exclusion code sets; 1:1 PSM.	Methods: Cohort Definitions
Variables	7	Outcomes and 29 matching covariates defined by standardized code concepts.	Methods: Covariates; Outcomes
Data sources/measurement	8	ICD-10-CM, SNOMED, CPT, VA drug class, lab concepts via TriNetX.	Methods: Data Source; Outcomes
Bias	9	PSM on 29 covariates; residual imbalance reported and discussed; associations not causal.	Methods; Discussion
Study size	10	All eligible network patients; matched samples 38,569 and 149,002 per arm.	Methods; Results
Quantitative variables	11	Risk computed within fixed windows (30/90/180/365 d).	Methods: Statistical Analysis
Statistical methods	12	Risk, RR, and RD with 95% CIs; outcome-before-window exclusion; nominal P values.	Methods: Statistical Analysis
Participants (results)	13	Pre- and post-match counts reported.	Results: Cohort characteristics; Figure 1; Table 2
Descriptive data	14	Baseline characteristics and SMDs.	Table 2; Table 3
Outcome data	15	Events and denominators per outcome/window.	Table 4
Main results	16	RRs (95% CI) for all outcomes and windows.	Results; Table 4; Figures 2-3
Other analyses	17	Secondary postprocedural-pain cohort and control dermatologic measures (Table 4); non-opioid composite disaggregation (Table 5); procedure-specific sensitivity analyses (Table 6).	Results; Table 4; Table 5; Table 6
Key results	18	Equal/greater pain but lower systemic analgesia; higher PONV.	Discussion (first paragraph)
Limitations	19	Observational aggregate EHR data; residual and unmeasured confounding, including unmatched procedure type and surgical severity; incomplete medication capture (over-the-counter or outside contributing organizations); heterogeneous exposure analyzed collectively with a composite non-opioid outcome; nonspecific PONV code (R11); and no validated pain severity, functional status, or patient-reported outcomes.	Discussion
Interpretation	20	Hypothesis-generating perioperative pain-management difference; not causal.	Discussion; Conclusions
Generalizability	21	Single federated network; external replication needed.	Discussion; Conclusions
Funding	22	None reported.	Reported in the journal submission system
